# The effect of aerobic and resistance exercise on the progression of colorectal cancer in an animal model

**DOI:** 10.1590/acb384923

**Published:** 2023-10-23

**Authors:** Marcelo Barbosa Neves, Udenilson Nunes da Silva, Alessandra de Figueiredo Gonçalves, Letícia Silva Fagundes, Antônio Carlos de Abreu, Luiz Carlos Takita, Ricardo Dutra Aydos, Rondon Tosta Ramalho

**Affiliations:** 1 Universidade Federal do Rio de Janeiro – Postgraduate Program in Biological Sciences – Rio de Janeiro (RJ) – Brazil.; 2 Universidade Federal do Mato Grosso do Sul – Health and Development Postgraduate Program – Campo Grande (MS) – Brazil.; 3 Universidade Federal do Mato Grosso do Sul – Health and Development in the Midwest Region – Campo Grande (MS) – Brazil.; 4 Universidade Federal do Mato Grosso do Sul – Medical School – Campo Grande (MS) – Brazil.

**Keywords:** Colorectal Neoplasms, Colitis-Associated Neoplasms, Exercise, Azoxymethane, Models, Animal

## Abstract

**Purpose:**

The aim of this study was to assess the effects of resistance and aerobic exercise on colorectal cancer (CRC) development in mice induced by azoxymethane (AOM) coupled with colitis.

**Methods:**

Forty animals induced with CRC were used, divided into five groups of eight animals each: sedentary; continuous aerobics; continuous anaerobic; aerobic PI; and anaerobic PI. AOM was administered to the animals in two doses of 10 mg/kg each over the course of two weeks, the first dose administered in the third week and the second administered in the fourth. For the colitis, three cycles of dextran sodium sulfate were administered for five days, separated by two weeks of water. The 14th week of the experiment saw the euthanasia, the removal of their colons, and the creation of microscopy slides for histological analysis.

**Results:**

Preneoplastic lesions developed in all five groups; there were no significant differences between them. However, in terms of inflammatory symptoms, mucosal ulceration was much more frequently in the exercise groups than in the sedentary group (p = 0.016). The number of polyps overall (p = 0.002), the distal region’s polyp development (p = 0.003), and the proximal region’s polyp development (p = 0.04) were all statistically different than sedentary group.

**Conclusions:**

The study discovered no significant difference in disease activity index scores between groups, but there was a significant difference in the number of polyps and the presence of mucosal ulceration in the colon.

## Introdution

One of the most prevalent malignant tumors in the world, colorectal cancer (CRC) refers to cancer that affects the colon, the rectus sigmoid junction, and the rectum. It is the second most prevalent form for women and the third most common type for men worldwide^1^,^2^. In 2040, there would be 3.2 million new CRC cases worldwide, according to the prediction^3^.

The CRC has a multifactorial etiology. Both genetic and environmental factors play a significant role in the development of CRC^2^. Bowel cancer is also increased by inflammatory bowel diseases such as chronic ulcerative colitis and Crohn’s disease^4^. Sedentary behavior favors several metabolic alterations and promotes a significant increase in the risk of CRC^5^. Although the underlying mechanisms have not been clarified, sedentary behavior favors several metabolic alterations and promotes a significant increase in the risk of CRC^6^. Sedentary behavior is the world’s fourth leading cause of death. Every year, approximately 3.2 million people die as a result of lack of physical activity^7^.

Regular physical activity, on the other hand, is associated with a reduction in CRC of 24 to 31%^8^. Mechanisms in which physical exercise alters the development of CRC are through its effects on improving the function of the immune system and decreasing chronic inflammation and antioxidant defense, positively modulating factors related to the development of this type of cancer^9^,^10^. Je et al.^11^ conducted a meta-analysis of prospective cohort studies that revealed a 25% reduction in CRC-specific mortality in practitioners of any activity or physical exercise at any level before diagnosis compared to those who were sedentary, confirming the association of physical exercise with better disease outcomes.

Although a relationship between physical exercise and a lower risk of CRC has been demonstrated by previous research, the underlying mechanisms are not fully understood. Several theories have been put forward, one of which is that exercise can help lower oxidative stress and chronic inflammation, two factors that are known to play a role in the development of CRC. Additionally, exercise has been shown to improve immune function, which may help lower the incidence of CRC. There is, however, little information on the effects of physical activity at different times and intensities, with most current studies focusing on a specific type of exercise. By examining the effects of aerobic and resistance exercise on the development of CRC in mice caused by azoxymethane (AOM) paired with colitis, the present study sought to fill this information gap.

## Methods

The study was carried out at the Laboratory of Experimental Carcinogenesis of the Postgraduate Program in Health and Development in the Midwest Region of Medical School of Universidade Federal de Mato Grosso do Sul (UFMS).

All steps and procedures were carried out in accordance with the ethical principles established by the National Council for the Control of Animal Experimentation and were filed with the Ethics Committee on Animal Use of the UFMS, under No. 1091/2019.

### Animals

Thirty-nine male inbred HRS/J mice, known as Hairless, were used as experimental model of infection. The animals were obtained from the central animal facility of the UFMS.

Mice were aged 4-5 weeks and had an initial weight of 25 g. The animals were kept in collective cages (dimension 40 × 35 × 17 cm), holding four animals/cage. Boxes were housed on the same shelf with a height of 1 meter from the floor and exposure to light in the same way as all the cages, at a temperature of approximately 25°C, with a light/dark cycle of 12 hours, receiving standard chow (Nuvital CR1) and water *ad libitum*. They were acclimated to laboratory conditions for 14 days before the experiment.

### Experimental design

**Figure 1:**
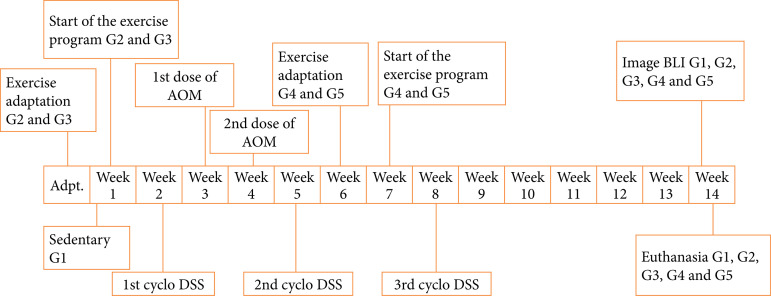
Experimental design: timeline.

AOM: azoxymethane; DSS: dextran sodium sulfate. Source: Elaborated by authors.

The experimental design is shown in Fig. 1. After a week of acclimatization, male mice were randomized into five groups (sedentary and exercised groups), divided as follows:

- G1 (sedentary): induced to CRC;
- G2 (aerobic continuous): continuous aerobic exercise before and after CRC induction;
- G3 (anaerobic continuous): continuous anaerobic exercise before and after CRC induction;
- G4 (aerobic PI): aerobic exercise after CRC induction;
- G5 (anaerobic PI): anaerobic exercise after CRC induction.

AOM was used as a carcinogen to induce CCR, and dextran sodium sulfate (DSS) to induce colitis by speeding up the carcinogenesis process.

### Physical exercise protocols

Swimming was chosen for aerobic exercise, and climbing a vertical ladder was chosen for anaerobic exercise. The animals were adapted six days before the protocols began, with the exercises performed gradually and on alternate days.

### Aerobic physical exercise

The animals were placed in groups of four in a tank with 29 L of heated water (31± 2°C), height of 27.6 cm and width of 33 cm, with the sessions being gradually increasing, adding 5 minutes of swimming each week, beginning with 10 minutes and ending with 30 minutes of swimming in the 14th week^12^.

### Anaerobic exercise

Resistance training on a vertical ladder involves the animals climbing the ladder with weights tied to their tails until they reach a compartment at the top. There were 10 repetitions of climbing the stairs to the top, with a 2-minute rest in between. Starting with a 10% overload of body weight, the load is increased by 10% every week until it reaches 50% in the 14th week^12^.

### Colitis and colorectal cancer induction

The mice were given water containing 2.5% DSS (MP Biomedicals, Santa Ana, CA, United States of America) for three cycles of seven consecutive days to induce colitis, interspersed with two weeks of normal water^12^.

For cancer induction, all animals received two intraperitoneal injections (right lower quadrant of the abdomen) of AOM (Sigma-Aldrich Laboratory) at the total dose of 20 mg/kg, divided into two weeks at 10 mg/kg per week^12^.

### Disease activity index

The disease activity index (DAI) was used to assess clinical symptoms such as body weight loss, stool characterization, and hematochezia. A researcher scored the DAI blindly^13^.

The presence of a tumor in the colon was determined by obtaining fluorescent optical images within the near-infrared (NIR) and X-ray images using the Laboratory of Studies in Experimental Models of Disease’s “In-Vivo Xtreme/Bruker II” system. The fluorescent biomarker IR-780 iodide Dye (Sigma Aldrich) was administered intraperitoneally to the animals at a dose of 0.334 mg/kg. After 12 hours, fluorescent NIR images were captured using a 760 nm excitation filter and an 830 nm emission filter^12^.

The presence of a tumor in the colon was determined by obtaining fluorescent optical images within the NIR and X-ray images using the Laboratory of Studies in Experimental Models of Disease’s “In-Vivo Xtreme/Bruker” system. The fluorescent biomarker IR-780 iodide Dye (Sigma Aldrich) was administered intraperitoneally to the animals at the dose of 0.334 mg/kg. After 12 hours, fluorescent NIR images were captured using a 760-nm excitation filter and an 830-nm emission filter^12^.

### Euthanasia

The animals were euthanized in the 14th week. They were given general inhalation anesthesia (isoflurane) before being exsanguinated. The absence of respiratory movement and heartbeat was considered to confirm the animals’ death.

### Collection and storage of colon

A stereoscopic microscope (JVC-Victor Company of Japan Lada Ltd., Model TK-C720EC) was used to examine the colons, identifying any abnormal changes, and counting the number of polyps^14^.

The suspect colon segments were placed in 10% buffered formalin for histological analysis as they were removed. Each colon segment was embedded in paraffin, cut with a microtome at a thickness of 5 μm, and stained with hematoxylin and eosin for further analysis by an experienced pathologist.

### Statistical analysis

The chi-square test, with Bonferroni correction when necessary, was used to assess the relationship between the experimental groups and the histological alterations, both of preneoplastic lesions or cancer and of inflammatory signs in the colon of the animals.

The one-way analysis of variance (ANOVA) test for variance with a Tukey-Kramer’s post-test was used to evaluate the number of polyps in the distal region and total polyps, as they had a normal distribution. Because the number of polyps in the proximal region did not have normal distribution, the Kruskal-Wallis’ test was used with a Dunn’s post-test.

The Statistical Package for the Social Sciences program, version 24.0, was used for the statistical analysis, with the significance level of 5%.

### Results

**Figure 2:**
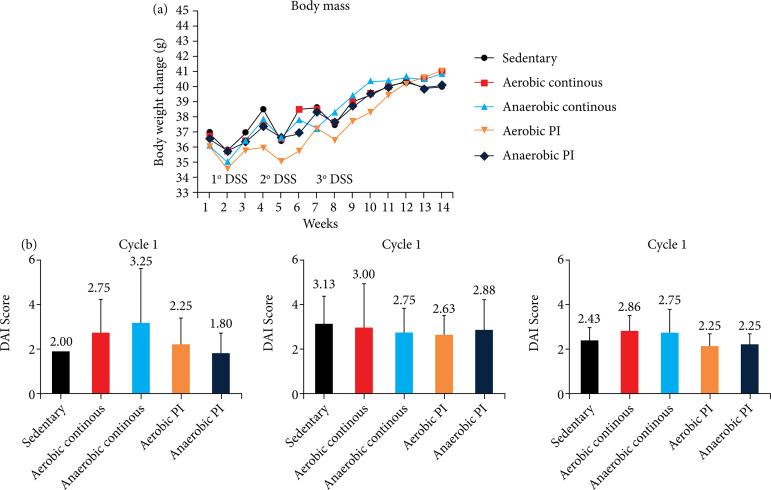
Effect of exercise on colitis-related symptoms. **(a)** Effects of physical exercise on body weight in mice. **(b)** DAI score. Data are reported as mean ± standard deviation of mean.

DAI: disease activity index; DSS: dextran sodium sulfate. Source: Elaborated by authors.

We assessed the effect of exercise on colitis-related symptoms, including the DAI score, and calculated the colitis severity score by adding weight loss, stool consistency change, and bleeding scores. Weight loss is a sensitive measure of colitis severity in the AOM/DSS-treated animal model, which is linked to carcinogenesis. There was no high and significant difference between the groups during the three DSS cycles (Fig. 2). Most of the animals had pasty stools, and in some animals with diarrhea, the test for occult blood revealed a higher presence in the second and third cycles in the *sedentary* group.

**Figure 3:**
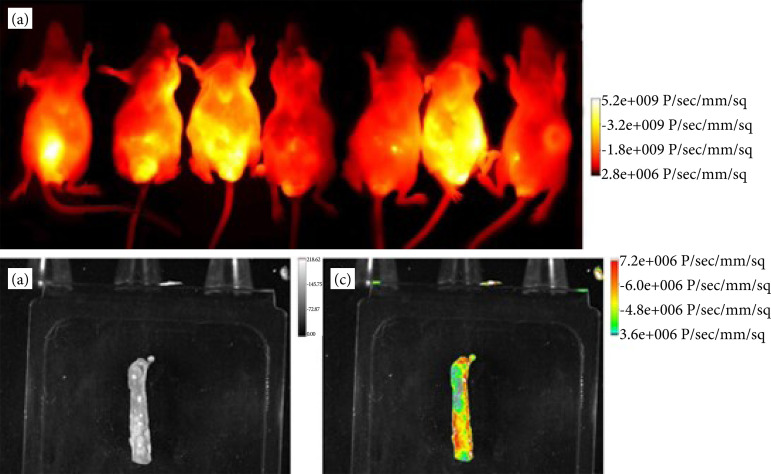
Images captured by the “In-VivoXtreme/Bruker II” system. **(a)** Images of the animals using a hot filter showing fluorescence in yellow. **(b)** Photo of a section of the distal colon. **(c)** Photo of a section of the colon superimposed by the fluorescence emitted by the polyps.

Source: Elaborated by authors.

The fluorescent marking in all analyzed groups was observed by evaluating the fluorescence in the NIR on the last day of the experiment, indicating tumor development in the colon of the animals (Fig. 3).

### Polyp developments

There was a significant difference in the comparison of experimental groups and number of polyps in the distal and total regions; we found no significant differences in the proximal region. The *sedentary* and *aerobic continuous* groups presented, respectively, 2.28 ± 1.11 and 2.28 ± 1.25 polyps, presenting a greater multiplicity when compared to the *anaerobic continuous*, *aerobic PI*, and *anaerobic PI* groups, which presented, respectively, 1.62 ± 074, 1.87 ± 1.12 and 1.00 ± 0.81 polyps, but this difference was not significant.

There was a difference in the distal region between the *aerobic PI*, *anaerobic continuous*, and *anaerobic PI* groups, which had 3.75 ± 1.48, 3.12 ± 1.12, and 3.62 ± 1.50 polyps, respectively, number significantly smaller (p = 0.003) than the *sendentary* group, which had 5.71 ± 1.89 polyps. When compared to the *sedentary* group, *aerobic continuous* with 6.14 ± 1.57 polyps did not show a significant difference.

**Figure 4:**
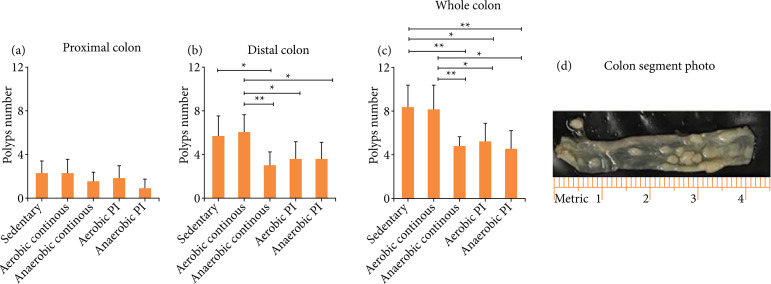
Effects of physical exercise on the multiplicity of polyps. After 14 weeks, the mean number of polyps and their distribution in the proximal, distal, and total colon in each group. **(a)** Proximal colon. **(b)** Distal colon. **(c)** Whole colon. **(d)** Colon segment photomicrograph.

In terms of total polyps, the *aerobic PI* group had 5.25 ± 1.66 polyps, *anaerobic continuous* had 4.75 ± 1.03 polyps, and *anaerobic PI* had 4.50 ± 1.77 polyps, which was significantly lower (p < 0.01) than the *sedentary group*, which had 8.42 ± 2.07 total polyps, and we also found a significant difference (p < 0.01) between the *aerobic PI*, *anaerobic continuous*, and *anaerobic PI* group when compared with *continuous aerobic*, that had 8,28 ± 2.21 total polyps (Fig. 4).

*p < 0.05; **p < 0.01. Source: Elaborated by authors.

### Histological analysis

**Figure 5:**
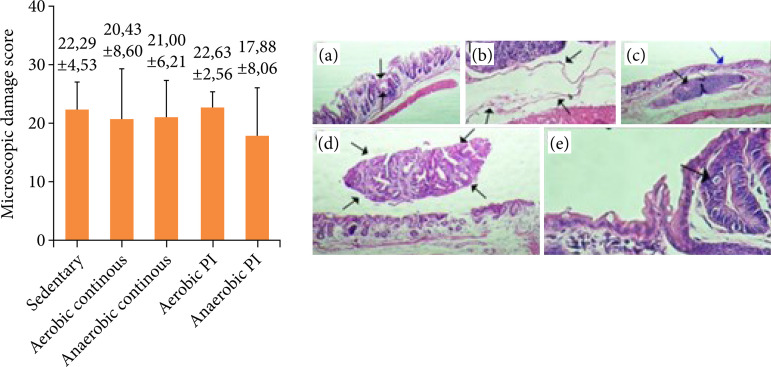
Microscopic damage score induced by azoxymethane/dextran sodium sulfate. Data are reported as mean±standard deviation of the mean. **(a)** Ulceration; **(b)** dilated lymphatic vessel; **(c)** black arrow: lymphoid plaque, blue arrow: lymphatic vessel with dilated lumen; **(d)** adenoma; **(e)** aberrant crypt.

Source: Elaborated by authors.

AOM/DSS caused epithelial and inflammatory lesions, leading to crypt formation and dysplasia, indicating the existence of inflammation and pre-neoplastic lesions (Fig. 5), along with increases in microscopic lesion scores. The score of microscopic damage shows that *aerobic continuous* (20.43 ± 8.60), *anaerobic continuous* (21.00 ± 6.21) and anaerobic PI (17.88 ± 8.06) mice had lower scores than *sedentary* (22.29 ± 4.53) and *aerobic PI* (22.63 ± 2.56), showing a possible effect of physical exercise in reducing inflammatory and preneoplastic lesions, but this difference was not significant.

There was no significant association between the groups regarding preneoplastic lesions such as aberrant crypts (*p*=0.905), adenomas (*p*=0.404), low-grade dysplasia (*p*=0.232) and high-grade dysplasia (Table 1).

**Table 1:** Results of histological changes, both pre-neoplastic/cancer lesions, and inflammatory signs in each of the experimental groups evaluated in this study*.

Histopathological changes		Sedentary (%) (n = 7)	Aerobic continuous (%) (n = 7)	Anaerobic continuous (%) (n = 8)	Aerobic PI (%) (n = 8)	Anaerobic PI (%) (n = 8)	P-Value
Pre-neoplastic/cancer lesions							
	Aberrant crypts	100 (7)	85.7 (6)	75 (6)	87.5 (7)	87.5 (7)	0.905
	Adenomas	100 (7)	85.7 (6)	62.5 (5)	75.0(6)	87.5 (7)	0.404
	Low-grade dysplasia	100 (7)	85.7(6)	75 (6)	100 (8)	100(8)	0.232
	High-grade dysplasia	0 (0)	0 (0)	0 (0)	0 (0)	0 (0)	-
Inflammatory signs							
	Discreet degree of mild inflammation	100 (7)	100 (7)	100 (8)	100 (8)	100 (8)	-
	Mucous ulceration	85.7 (6)ab	28. 57 (2)b	37.5 (3)ab	100 (8)a	62.5 (5)ab	0.016
	Reactive lymphoid tissue	85.7 (6)	85.7 (6)	87.5 (7)	100 (8)	87.5 (7)	0.612
	Crypt atrophy	100 (7)	71.45)	87.5(7)	100(8)	100(8)	0.176
	Dilation of lymph vessels	85.7 (6)	85.7 (6)	87.5 (7)	100(8)	87.5 (7)	0.872

* The results are presented in relative frequency (absolute frequency). The p-value in the chi-square test is reported. Different letters in the same row indicate significant differences between the experimental groups (chi-square test with Bonferroni correction). Source: Elaborated by authors.

On the other hand, regarding inflammatory signs, there was a significant association between the experimental groups and the presence of mucosal ulceration in the animals’ colons (*p* = 0.016), and the percentage of animals that presented these ulcerations in the *anaerobic PI* (100%) was significantly higher (*p* < 0.05) than those in *aerobic continuous* animals (28%). However, there was no significant association between the experimental group and inflammatory signs, such as the degree of mild inflammation, reactive lymphoid plaque (*p* = 0.612), crypt atrophy (*p* = 0.176), and lymphatic vessel dilation (*p* = 0.876).

## Discussion

In previous results, already published^12^, it was observed that aerobic exercise for seven weeks after tumor induction (*aerobic PI*) had a smaller impact on animal behavior. However, when the exercise was applied for 14 weeks, before and after tumor induction (*aerobic continuous*), we found a significant increase in the stress of these animals, although there was no difference in behavior between the resistance exercise groups, pre- and post-induction (*continuous anaerobic*) or just post-induction (*anaerobic PI*). Nonetheless, there was a slight improvement in stress/anxiety-type behavior reduction when compared to the *sedentary* group. It is worth noticing that the *aerobic continuous* group, which showed a significant increase in animal stress, also had a significant difference in the number of polyps compared to the other exercise groups in this study.

Understanding the benefits of regular physical activity on CRC is difficult because the beneficial results can vary depending on the type of activity, duration, and intensity^15^. Because of the different frequencies and mechanical loads imposed on the muscle, aerobic and resistance exercise can activate distinct molecular pathways^16^. Different intensities and durations of physical activity result in different metabolic adaptations, resulting in increased mitochondrial mass, oxygen supply, glucose uptake, and antioxidant capacity^17^. Physical exercise has a variety of physiological effects that can be classified as acute, late acute, or chronic depending on the intensity and modality^18^.

According to a study conducted by Lunz et al.^19^, different intensities of swimming exercise did not reduce the development of CRC when compared to the control group; only the 2% intensity protected against preneoplastic lesions of the colon induced by dimethylhydrazine (DMH), but not against tumor development. This finding is consistent with our own, in which the aerobic continuous group had more polyps than the other exercised and sedentary groups. This finding indicates that the intensity and duration of exercise influence the frequency and number of preneoplastic lesions.

Exhausting physical activity can contribute to the occurrence and multiplicity of preneoplastic lesions. According to Demarzo and Garcia^20^, a single session of exhaustive exercise increased the number of aberrant colonic crypts in rats treated with DMH compared to control group animals. According to our findings, the swimming duration in the *aerobic continuous* group may have been exhausting, resulting in an increase in the number of polyps. Exhaustive physical activity can result in oxidative stress, which is caused by an imbalance between the production of reactive oxygen species (ROS) and insufficient antioxidant defense and causes cell and tissue damage, which can result in epigenetic changes that initiate or enhance cancer development^21^,^22^.

Reddy et al.^23^ demonstrated in their study that when animals exercised in a voluntary wheel compared to sedentary animals the exercise had no effect on the incidence and multiplicity of colonic adenomas. These findings were consistent with ours, in which the groups of animals that exercised compared to the sedentary group had no significant difference in the number of adenomas.

Exhaustive swimming exercises cause tissue damage regardless of training status by increasing muscle lipid peroxidation and causing the loss of mitochondrial integrity and reactive species found in exhausted trained and untrained rats^24^. Exhausting physical activity can cause oxidative stress, which is caused by an imbalance between ROS production and inadequate antioxidant defense. This negative condition can cause cell and tissue damage and is involved in a variety of pathophysiological states such as aging, exercise, inflammatory, cardiovascular, and neurodegenerative diseases, and cancer^21^,^22^.

While aerobic training reduced the number of dysplastic lesions of the colon by 36% compared to the sedentary group exposed to carcinogenic agents in a study by Colbert et al.^24^, there was no significant difference in the incidence of preneoplastic lesions in our study. Voluntary exercise before or during AOM exposure resulted in a significant reduction in the number of polyps in the study by Kelly et al.^25^, but exercise after AOM exposure had no effect. In our study, however, the aerobic PI and anaerobic PI groups had significantly fewer total polyps than the aerobic continuous group and the sedentary group.

Matsuo et al.^26^ found that high-intensity intermittent swimming reduced the number of aberrant crypts generated by DMH in the rat colon, implying that high-intensity physical training may be beneficial in the prevention of colon cancer. Fuku et al.^27^ discovered that low-intensity running training prevents the DMH-induced development of aberrant crypt foci in the colon. Both studies found that different types and intensities of physical activity can cause different responses in neoplastic lesions.

Chronic inflammation has been linked to the development of many cancers, including colon cancer. Inflammation in the intestine promotes carcinogenic mutagenesis and the onset of CRC^28^. The inflammatory environment is very similar to the tumor microenvironment, implying that the same mediators are involved in chronic intestinal inflammation and colorectal carcinogenesis, and many inflammatory mediators have been linked to an increase in the prevalence of colorectal adenomas^29^,^30^. We found significant differences in mucosal ulceration between the *aerobic continuous* group and the *aerobic PI* group when we examined colon tissue for inflammatory signs consistent with injury to the mucosa of the gastrointestinal tract.

Qin et al.^31^ used a model that was similar to ours in the rats. They were induced with chronic colitis by DSS and subjected to a seven-week swimming program of 90 minutes per day, five days per week. Swimming reduced the production of neutrophil pro-inflammatory cytokines and chemokines, as well as tumor necrosis factor-ɑ and interferon-y, and decreased the protein expression of nuclear phosphorylated factor-B, p65, and cyclooxygenase 2, while increasing interleukin-10 levels, demonstrating that physical exercise has an anti-inflammatory effect on colon tissue.

Our study used an animal model of CRC, which might not accurately reflect the many molecular and physiological variables involved in the development of CRC in humans. So, when applying the results to clinical practice, care should be taken. Another limitation is that our study did not evaluate specific oxidative stress and inflammatory cytokine indicators. Our work concentrated on the emergence of colorectal preneoplastic lesions, although these measurements would have offered a more thorough knowledge of the underlying mechanisms at play. Future research could benefit from extended follow-up periods to monitor disease progression to more advanced stages and from the analysis of inflammatory cytokines to better clarify potential relationships between physical activity and CRC carcinogenesis.

Despite these limitations, our study provides important knowledge regarding the possible effects of exercise on CRC and points out areas that require more research.

## Conclusion

The study discovered no significant difference in DAI scores between groups, but there was a significant difference in the number of polyps and the presence of mucosal ulceration in the colon. The group that did continuous anaerobic exercise before and after CRC induction had a significant increase in animal stress and the number of polyps when compared to the other exercise groups. Depending on the type, duration, and intensity of the activity, physical exercise can have varying effects on CRC. More research is required to understand the mechanisms underlying these effects.

## Data Availability

The data will be available upon request.
